# First Record of the Genus *Cartorhynchites* Voss, 1958 (Coleoptera: Rhynchitidae) from Eocene Baltic Amber with a List of Fossil Tooth-Nosed Snout Weevils

**DOI:** 10.3390/life13091920

**Published:** 2023-09-15

**Authors:** Andrei A. Legalov, Andris Bukejs, Anarina Vanaga, Vitalii I. Alekseev

**Affiliations:** 1 Institute of Systematics and Ecology of Animals, Siberian Branch, Russian Academy of Sciences, 630091 Novosibirsk, Russia; fossilweevils@gmail.com; 2 Department of Ecology, Biochemistry and Biotechnology, Altai State University, 656049 Barnaul, Russia; 3 Department of Forestry and Landscape Construction, Tomsk State University, 634050 Tomsk, Russia; 4 Institute of Life Sciences and Technologies, Daugavpils University, Vienıbas 13, 5401 Daugavpils, Latvia; 5 Kaliningrad Regional Amber Museum, Marshal Vasilevskii Square 1, 236016 Kaliningrad, Russia; alekseew0802@yahoo.com; 6 Immanuel Kant Baltic Federal University, Nevskogo Str. 14, 236016 Kaliningrad, Russia

**Keywords:** paleobiodiversity, Curculionoidea, new species, host plants, Cenozoic, X-ray micro-CT

## Abstract

A new species of the genus *Cartorhynchites* (Rhynchitini, Rhynchitina) is described from Baltic amber. *Cartorhynchites groehni* Legalov, Bukejs et Alekseev sp. n. differs from *C*. *struvei* Zherikhin, 1992 from the Miocene of Germany in its smaller body size (2.6 mm), strongly convex eyes, narrower pronotum and wide elytra, and dark brown legs. A new species is studied and illustrated in detail using X-ray micro-computed tomography (μCT). It is the earliest fossil record of subtribe Rhynchitina. A list of fossil Rhynchitidae was compiled. A key to species of Rhynchitidae in Baltic amber was given. Fossil finds of the family Rhynchitidae were discussed. The assumption was made that the Recent distribution range of the genus *Cartorhynchites* is within the range of its host plant of the genus *Symplocos*. Probably, a new Eocene species developed on *Symplocos kowalewskii,* and the Oligocene *C*. *struvei* was associated with *Symplocos myosotis* (Unger).

## 1. Introduction

Curculionid beetles are a diverse group, numerous in fossil and modern ecosystems. This group includes approximately 62,000 described species [1] and is characterized by a complex taxonomic structure. There is no consensus on how many families this superfamily comprises [1,2,3,4,5,6,7,8,9,10]. Curculionoidea is represented by 131 extinct species in Baltic amber (e.g., [9,11,12,13,14,15,16,17,18,19,20,21,22,23,24,25,26,27,28,29,30,31,32,33,34]).

The family Rhynchitidae, often considered part of the family Attelabidae, was also found in Eocene amber [9,16,25,27,28,31,32,33,34]. Adults are characterized by mandibles externally dentate with which they gnaw plants’ vegetative or generative organs for the development of their larvae; some groups roll packets of leaves [35,36,37]. Previously, all tooth-nosed snout weevils found in Baltic amber belonged only to primitive groups such as the extinct tribe Sayrevilleini, the modern tribe Auletini, and the subtribes Temnocerina and Perrhynchitina from Rhynchitini [33]. Representatives of advanced groups (Deporaini, Byctiscini, and Rhynchitina of Rhynchitini) have not been described from Baltic amber.

This article describes a new extinct species of the genus *Cartorhynchites* Voss, 1938. A three-dimensional model reconstructed from μCT data was used to assist in the description of the new species. This is not only the first record of a representative of the subtribe Rhynchitina in Baltic amber but also the earliest find of this group in the fossil record. A list of fossil tooth-nosed snout weevils and a key to Rhynchitidae from Baltic amber are compiled.

## 2. Materials and Methods

The paleontological material examined is deposited in the collection of Carsten Gröhn (Glinde, Germany) [CCGG], separately deposited in the Center of Natural History (Centrum für Naturkunde–CeNak; formerly the Geological-Paleontological Institute and Museum–das Geologisch-Paläontologische Museum) [GPIH] of the University of Hamburg, Germany. The amber piece was polished manually with emery papers of different grit sizes, allowing improved views of the included specimen. The amber piece was not subjected to any supplementary fixation.

The X-ray micro-CT (μCT) observations of specimen “5098” [GPIH] were conducted at the Daugavpils University, Daugavpils, Latvia, using Zeiss Xradia 510 Versa system. Scans were performed with a polychromatic X-ray beam at energy of 40 kV and power of 3W. The sample–detector distance was set to 38 mm, and source to sample distance was 46 mm. Tomographic slices were generated from 2401 rotation steps through a 360-degree rotation, using a 4× objective, and exposure time during each projection was set to 12 s. Acquired images were binned (2 × 2 × 2), giving a voxel size of 3.69 μm. Prior to each full scan, a 20 min warm-up scan was conducted with identical stitch parameters but with reduced rotational steps (201 steps) and exposure times set to 2 s. Images were imported into Dragonfly PRO (ver. 2022.2) software platform for interactive segmentation, data alignment and stitching, 3D visualization, and producing videos of scan data (Supplementary Materials Videos S1–S3).

The photographs of the specimen were taken using a Canon 90D camera with a macro lens (Canon MPE-65 mm). Extended depth of field at high magnifications was achieved by combining multiple images from a range of focal planes using Helicon Focus v. 6.0.18 software, and the resulting images were edited to create figures using Adobe Photoshop CS5.

Specimen observations were made using a Nikon SMZ 745T stereomicroscope. Measurements of the holotype were made using the 3D reconstruction dataset within Dragonfly PRO.

We used previous reports from the literature [37,38,39,40,41,42,43,44,45,46,47,48] and collection data from the Institute of Systematics and Ecology of Animals, Siberian Branch, Russian Academy of Sciences (Novosibirsk, Russia), the Natural History Museum (London, UK), Hungarian Natural History Museum (Budapest, Hungary), Institut Royal des Sciences Naturelles de Belgique (Brussels, Belgium), National Museum of Natural History (Prague, Czech Republic), Zoological Institute of Russian Academy of Sciences (St. Petersburg, Russia), and Museum für Naturkunde der Humboldt-Universität (Berlin, Germany) to show the Recent distributions and localities of fossil forms of the genus *Cartorhynchites*.

Fossil Rhynchitidae have been described and recorded from 11 localities:

New Jersey amber—United States: Central New Jersey, near the town of Sayreville, South Amboy Fire Clay, Raritan Formation; Upper Cretaceous, Turonian, 93.9–89.8 ± 0.3 Ma;

Orapa—Botswana: Central Botswana, Orapa diamond mine; Upper Cretaceous, Turonian, 91 Ma;

Menat—France: Puy-de-Dome, Middle-Upper Paleocene, Selandian-Thanetian, 61.0–59.0 Ma;

Green River—United States: Colorado, 3–4 km west of railway crossing of Green River, Green River Formation; Lower Eocene, Ypresian, 50.6–48 Ma;

Baltic amber—Russia: Kaliningrad Oblast, Baltic Sea coast and Primorsky amber quarry 2 km east of Yantarny village, Prussian Formation, Middle–Late Eocene, Bartonian–Priabonian, 41.3–33.9 Ma;

Florissant—United States: Colorado, Rocky Mountains, near Pike’s Peak, Florissant Formation; Uppermost Eocene, Priabonian, 34.07 ± 0.10 Ma.

Rott—Germany: Nordrhein-Westfalen, Siebengebirge, near Bonn, Rott Formation; Uppermost Oligocene, Upper Chattian, 24.0–23.0 Ma;

Enspel—Germany: Rheinland-Pfalz, Westerwald, Bad Marienberg; Upper Oligocene, Upper Chattian, 24.79–24.56 Ma;

Dominican amber—Dominican Republic: mines in the Cordillera Septentrional, Lower Miocene, Burdigalian, 20–16 Ma;

Mexican amber—Mexico: Yukatan, Chiapas; Sinojovel Formation; Lower Miocene, Burdigalian, 20–16 Ma;

Öhningen (=Oeningen)—Germany: Baden-Württemberg, near Constance Lake (=Bodensee); Upper Freshwater Molasse Formation; Upper Miocene, Langhian-Tortonian (Sarmatian), 15–11.1 Ma;

Binagady—Azerbaijan: Binagady District, 7 km northwest of Baku, 0.5 km southeast of Binagady, Binagady tar pits; Quaternary, Middle Pleistocene; Odintsovo (=Korshov, =Lublinian) interglaciation, 0.190 Ma.

The morphological terminology used in this paper follows Lawrence et al. [49].

The systematics of studied taxa is based on the works of A. Legalov [9,10,37].

Nomenclatural acts introduced in the present work are registered in ZooBank (www.zoobank.org accessed on 8 September 2023) under LSID urn:lsid:zoobank.org:pub:C7B2216C-C3A9-44D7-ADCC-E9EB2847708F.

## 3. Results

*Systematics*

Superfamily **Curculionoidea** Latreille, 1802

Family **Rhynchitidae** Gistel, 1848

Subfamily **Rhynchitinae** Gistel, 1848

Supertribe **Rhynchititae** Gistel, 1848

Tribe **Rhynchitini** Gistel, 1848

Subtribe **Rhynchitina** Gistel, 1848

Genus ***Cartorhynchites*** Voss, 1938

Subgenus ***Hyperinvolvulus*** Legalov, 2003

Type species: *Cartorhynchites angusticlavus* Legalov, 2003

**Figure 1:**
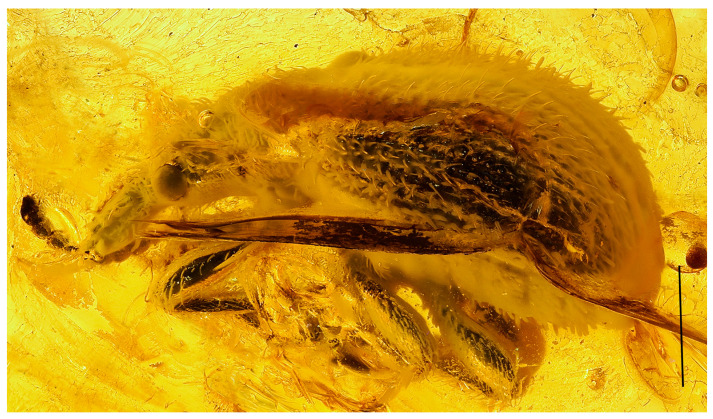
Photomicrograph of *Cartorhynchites* (*Hyperinvolvulus*) *groehni* sp. n., holotype, No. 5098 [GPIH], habitus in lateral view. Scale bar represents 1.0 mm.

**Figure 2:**
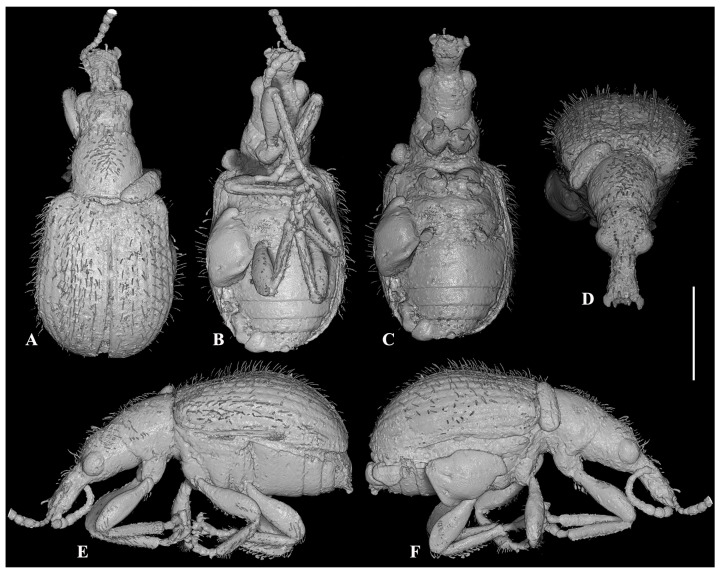
X-ray μCT renderings of *Cartorhynchites* (*Hyperinvolvulus*) *groehni* sp. n., holotype, No. 5098 [GPIH], habitus. (**A**) dorsal view; (**B**) ventral view; (**C**) idem, with legs and antennae removed; (**D**) frontal view; (**E**) left lateral view; (**F**) right lateral view. Scale bar represents 1.0 mm.

**Figure 3:**
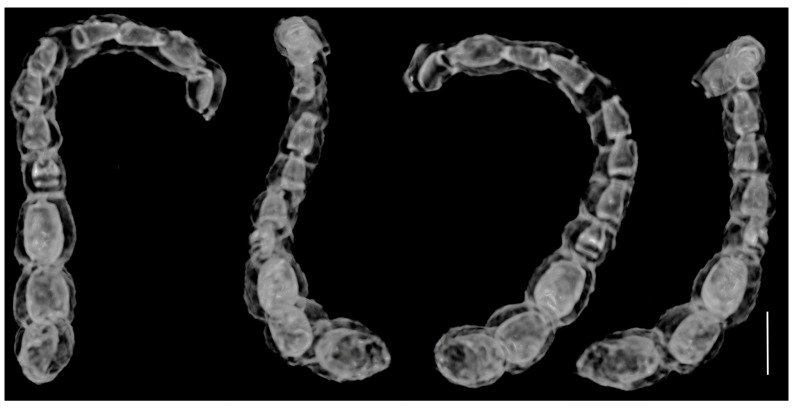
X-ray μCT renderings of *Cartorhynchites* (*Hyperinvolvulus*) *groehni* sp. n., holotype, No. 5098 [GPIH], right antenna in different views. Scale bar represents 0.1 mm.

***Cartorhynchites* (*Hyperinvolvulus*) *groehni*** Legalov, Bukejs et Alekseev **sp. n.** (Figure 1, Figure 2 and Figure 3), (Supplementary Materials Videos S1–S3)

LSID: urn:lsid:zoobank.org:act:3D343D7E-2575-4245-9234-67E90B8E0242.

**Type stratum**. Baltic amber; Middle–Late Eocene [50,51,52,53,54].

**Type locality**. Yantarny village (formerly Palmnicken), Kaliningrad Region, Russia.

**Derivation of name**. The specific epithet is a patronym and is dedicated to Carsten Gröhn (Glinde, Germany), an enthusiast and specialist in Baltic amber.

**Diagnosis**. The new species differs from *C*. *struvei* Zherikhin, 1992 from the Miocene of Germany in its smaller body sizes (2.6 mm), strongly convex eyes, narrower pronotum and wide elytra, and dark brown legs. It is distinguished From other Baltic amber Rhynchitini by its elytra lacking scutellar striole and its rostrum being shorter than its pronotum.

**Description**. Measurements: body length (without rostrum) about 2.56 mm, body maximum width 1.47 mm; rostrum length 0.62 mm; head length 0.43 mm and head width (across eyes) 0.63 mm; pronotum length 0.72 mm and pronotum maximum width 0.75 mm; and elytra length 1.89 mm and elytra maximum combined width 1.47 mm.

Body convex, dark brown (as preserved). Pubescence: head, pronotum, and elytra with rather dense and long, semierect to erect setae; ventral side of body and legs with shorter, recumbent to semierect setae.

The head has a flat forehead, convex vertex, and large temples about as long as one eye length. Rostrum is quite thick, rather short, 0.86× as long as pronotum, almost straight in lateral view, widest apically, about 2.0× as long as wide basally, 1.8× as long as wide medially, 1.4× as long as wide apically, and is covered with very fine and sparse punctures. The compound eyes are large, subhemispherical, and strongly convex, with a vertical diameter of about 0.88× the horizontal diameter. The mandibles are externally dentate. The maxillary palpi apparently have four palpomeres.

The Antennae have 11 antennomeres, not geniculate, with a distinct three-antennomered club; they are rather long, about 1.36× as long as the rostrum, reaching the middle of the pronotum, inserted laterally in basal one-third of rostral length. The scape is cylindrical, 1.5× as long as it is wide; antennomere 2 is subcylindrical, equal in size as the scape, and 1.3× as wide as antennomere 3; antennomeres 3–4 are conical, elongate, 2.2× as long as they are wide, and equal in size; antennomere 5–8 are conical, subequal in width, 1.2–1.4× as long as they are wide, and slightly shorter than antennomere 4; the antennal club (antennomeres 9–11) is about 0.7× as long as the flagellum (antennomeres 2–8); antennomere 9 is suboval, about 1.3× as long as it is wide, slightly dilated apically, and about 1.5× as wide as antennomere 8; antennomere 10 is suboval, slightly dilated apically, about 1.2× as long as it is wide, and slightly wider than antennomere 9; and antennomere 11 is ovoid, 1.6× as long as it is wide. The relative length ratios of antennomeres 1–11 are equal to 12:12:13:13:10:10:10:10:16:16:23.

The pronotum is nearly bell-shaped, widest in the middle, slightly narrowed posteriad and greatly narrowed anteriad, nearly as long as it is maximum wide, and moderately densely covered with small punctations; its disc is slightly convex, lateral margins are widely rounded medially and oblique anteriorly and posteriorly, posterior margin is convex, and anterior margin is convex from the dorsal view.

The scutellum is nearly pentagonal and is about 0.8× as long as its maximum width.

The Elytra are widely oval, convex, elongate, widest in the middle and 1.3× as long as they are wide combined, 1.5× as long as they are wide combined at the anterior margin, 1.4× as long as they are wide combined in the posterior one-quarter, and 2.6× as long as the pronotum length; the elytral base is emarginated and distinctly wider than the posterior pronotal margin; setae spots are absent; the humeral callus is distinct and prominent; and each elytron is widely rounded separately. The elytral punctations are rather coarse and dense, apparently forming 10 regular rows; the distance between punctures in rows is smaller than the diameter of the punctures; punctures do not merge and do not form pits; and scutellary strioles are absent. Stria 9 is shortened and connected with stria 10 near the metacoxal cavity; interstriae are convex, and the distance between rows is about 1.0× the diameter of the punctures; the apices of the elytra are separately rounded. The elytral epipleuron is wide; it is widest at the humeri and gradually narrows posteriad.

The pre- and postcoxal parts of the prosternum are short. The precoxal part is about 0.05× as long as the procoxal cavity length. The postcoxal part is 0.2× as long as the procoxal cavity length and about 3.7× as long as the precoxal part. The metaventrite with disc convex is about 0.8× as long as the metacoxal cavity length. The metepisternum is about 7.0× as long as wide in the middle. The abdomen is convex and covered with small punctations, with five visible ventrites. Ventrites 1 and 2 are fused, and the suture between ventrites 1 and 2 is indistinct; the sutures between ventrites 2 and 5 are complete and almost straight; ventrite 1 is slightly longer than the metaxocal cavity length; ventrite 5 has a widely rounded apical margin; and the relative length ratios of ventrites 1–5 equal to 6.2:6.8:4.7:3.5:3 (medially). The propygidium is concealed by the elytra. The pygidium is exposed.

The legs are long and slender. The procoxae are conical, contiguous, and located in the middle of the prosternum; the mesocoxae are widely oval, transverse, about 1.3× as wide as they are long, and separated by 0.25× the width of the mesocoxa; the metacoxae are suboval, transverse, and reach the metepisternum. The femora clavate is slightly flattened, covered with fine, sparse punctations, and about 3.0× as long as it is maximum wide. The tibiae are cylindrical, straight, about 6.1× as long as they are maximum wide, nearly as long as the femora, and have sparse and fine punctures. They are not serrated on the inner margin and have a fringe of spinulae apically, without uncus and mucro. The tarsi are long but shorter than the tibiae; the metatarsus is about 0.6× as long as the metatibia. Tarsomere 1 is subconical, slightly dilated apically, and elongate; tarsomere 2 is trapezoidal, nearly as long as it is wide; tarsomere 3 is deeply bilobed, as long as it is wide; tarsomere 4 (apical tarsomere) is subcylindrical, elongate, and slightly curved; metatarsomere 1 is about 1.9× as long as it is maximum wide, slightly shorter than tarsomere 4, 1.9× as long as tarsomere 2, and about 0.4× as long as tarsomeres 2–4 combined; metatarsomere 2 is about 1.2× as long as it is maximum wide; metatarsomere 3 is as long as it is maximum wide; metatarsomere 4 is about 2.6× as long as it is maximum wide; and the relative length ratios of metatarsomeres 1–4 are subequal to 15:8:10:18. The Tarsal claws are free, apparently have teeth basally, and are slightly divergent.

**Material examined**. Holotype: collection number “5098” [GPIH], “8700” [CCGG] (ex. coll. Jonas Damzen JDC-10503); adult, female. A complete beetle is included in a transparent, yellow amber piece with dimensions of 24 × 15 mm and a maximum thickness of 5 mm and is preserved without supplementary fixation. The right part of the specimen is completely obscured by milky amber. Syninclusions: two larvae of mites (Araci), a few stellate Fagaceae trichomes, and numerous small gas vesicles. The sex of the examined specimen was determined based on micro-CT results. There is no sclerotized aedeagus-like structure present inside the abdomen, and therefore, the specimen appears to be female.

**Remarks**. The specimen under consideration possesses the combination of characters corresponding to the family Rhynchitidae: antennae that are not geniculate, a distinct epipleuron, maxillary palpi with four palpomeres, ventrites 1 and 2 being fused, tarsomere 1 not being extended, claws that are free at the base, and tibiae that are not serrated on the inner margin. This fossil specimen can be classified into the subfamily Rhynchitinae based on slightly divergent tarsal claws. Externally dentate mandibles suggest placement in the supertribe Rhynchititae. The new species belongs to the tribe Rhynchitini based on contiguous procoxal cavities, separately rounded apices of the elytra, a propygidium concealed by the elytra, metacoxa reaching the metepisternum, convex eyes, and striate elytra. Elytra lacking scutellar strioles suggest placement in the subtribe Rhynchitina. The new species belongs to the genus *Cartorhynchites* based on a quite thick rostrum that is shorter than the pronotum, a quite short antennal club that is shorter than the flagellum, setae spots on the elytra being absent, punctures in the elytral striae that do not merge and do not form pits, and the eyes being strongly protruding. The dark body covered with long semierect to erect setae suggests placement in the subgenus *Hyperinvolvulus*.

**Key to species of Rhynchitidae in Baltic amber**

1. Tarsal claws strongly divergent and lacking teeth ([10] (Figure 62)) (*Baltocar*, Sayrevilleinae) ..…………………………………..……………………..………………..……………….2

– Tarsal claws are slightly divergent ([10] (Figure 61)) and as is the rule with teeth, if lacking teeth, then the rostrum is longer than the pronotum (Rhynchitinae)…………......7

2. Rostrum shorter or subequal to pronotum. Elytra with irregular punctations ([9] (pl. 7, Figure 1)) ……..…………………………………………………………………………….……..3

– Rostrum longer or subequal to pronotum. Elytra with punctations forming regular rows ([9] (pl. 7, Figure 2)) ……….………………………………………………………………4

3. Rostrum slightly curved ([9] (pl. 7, Figure 1)), subequal to pronotum. Body covered with dense………..………………………………………..…………………..………*B*. *convexus*

– Rostrum distinctly curved ([25] (Figure 55)), shorter than pronotum. Body without distinct pubescence …………………………………………………………………….*B*. *subnudus*

4. Pronotum coarsely punctate. Setae widened………………………………….*B*. *succinicus*

– Pronotum transversely irregularly rugose. Setae narrow……………………..…………..5

5. Pronotum with slightly rounded lateral sides ([25] (Figure 28)), about 1.1× as long as wide. Tarsomere 1 is about 0.6× as long as tarsomeres 2–5 combined ….……………. ……………………………………………………………………………………...*B*. *hoffeinsorum*

– Pronotum with subparallel lateral sides ([25] (Figure 21)), 1.4× as long as it is wide. Tarsomere 1 is about 0.4× as long as tarsomeres 2–5 combined …………………………….6

6. Rostrum long ([25] (Figure 16)), 18.0× as long as it is wide medially. Tarsomere 1 is slightly longer than tarsomere 5 and 1.2× as long as tarsomere 2. Elytra 1.2× as long as their maximum combined width. Body longer (2.9 mm).…………………………..*B*. *groehni*

– Rostrum shorter ([27] (Figure 1)), 6.8× as long as it is wide medially. Tarsomere 1 is shorter than tarsomere 5 and 1.4× as long as tarsomere 2. Elytra 1.5× as long as their maximum combined width. Body shorter (2.4 mm)…….………………..……….*B*. *sontagae*

7. Rounded apex of elytra when both together. (Auletini).…………...……………………..8

– Apices of elytra separately rounded (Rhynchitini).……………………………………… 11

8. Tarsal claws lacking teeth (*Electrauletes*, Auletini)………………………………...*E*. *unicus*

– Tarsal claws with teeth…………………………………………..…………………………….9

9. Rostrum very long ([27] (Figure 3)), 10.5× as long as wide at middle Tibiae lacking costate dorsal margin (*Pseudomesauletes*, Pseudomesauletina) ….…….………....*P*. *lobanovi*

– Rostrum 4.6-7.3× as long as wide at middle ([28] (pl. 6, Figure 4); [32] (Figure 1)). Tibiae with costate dorsal margin (Pseudauletina) …………………………………………………10

10. Body covered with dense protruding setae. Rostrum dorsally flattened, 7.3× as long as wide at middle (*Pseudauletes*)………….….………………………………………...…*P*. *balticus*

– Body almost glabrous. Rostrum dorsally not flattened, 4.6× as long as wide at middle (*Eoropseudauletes*)……………….………………………………………………...…..*E*. *plucinskii*

11. Elytra lacking scutellar striole. Rostrum shorter than pronotum (Figure 2) (*Cartorhynchites*, Rhynchitina)…………………………….……….………………..*C*. *groehni* sp. n.

– Elytra with scutellar striole. Rostrum longer than pronotum…..…………….…………..12

12. Body black, without bronze luster. Rostrum 2.7× as long as pronotum, about 14× as long as wide at middle ([30] (Figure 1)) (*Eocenorhynchites*, Temnocerina) …….………………………………………………………………………………………... *E*. *vossi*

– Body black, with bronze luster. Rostrum 1.1× as long as pronotum, about 5× as long as wide at middle ([31] (Figures 17 and 18)) (*Succinorhynchites*, Perrhynchitina) .…….... ………………………………………………………………………………………….….*S*. *alberti*

**List of fossil Rhynchitidae**

Subfamily **Sayrevilleinae** Legalov, 2003

Tribe **Sanyrevilleini** Legalov, 2003

Genus ***Orapauletes*** Legalov, 2009

*O*. *cretaceus* Legalov, 2009 [55]–Orapa, Botswana, Turonian, 91 Ma

Genus ***Sanyrevilleus*** Gratshev et Zherikhin, 2000

*S*. *grimaldii* Gratshev et Zherikhin, 2000 [56]–New Jersey amber, USA, Raritan Formation, Turonian, 93.9–89.8 ± 0.3 Ma

Genus ***Baltocar*** Kuschel, 1992

*B*. *convexus* Legalov, 2015 [9]–Baltic amber, Russia, Prussian Formation, Middle–late Eocene, Bartonian–Priabonian, 41.3–33.9 Ma

*B*. *groehni* Riedel, 2012 [25]–Baltic amber, Russia, Prussian Formation, Middle–late Eocene, Bartonian–Priabonian, 41.3–33.9 Ma

*B*. *hoffeinsorum* Riedel, 2012 [25]–Baltic amber, Russia, Prussian Formation, Middle–late Eocene, Bartonian–Priabonian, 41.3–33.9 Ma

*B*. *sontagae* Bukejs et Legalov, 2021 [27]–Baltic amber, Russia, Prussian Formation, Middle–late Eocene, Bartonian–Priabonian, 41.3–33.9 Ma

*B*. *subnudus* Riedel, 2012 [25]–Baltic amber, Russia, Prussian Formation, Middle–late Eocene, Bartonian–Priabonian, 41.3–33.9 Ma

*B*. *succinicus* Voss, 1953 [16]–Baltic amber, Russia, Prussian Formation, Middle–late Eocene, Bartonian–Priabonian, 41.3–33.9 Ma

Tribe **Vossicartini** Legalov, 2003

Genus ***Germanocartus*** Legalov, 2007

*G*. *heydeni* (Schlechtendal, 1894) [57]–Rott, Germany, Rott Formation, Upper Chattian, 24.0–23.0 Ma

Subfamily **Rhynchitinae** Gistel, 1848

Supertribe **Rhynchititae** Gistel, 1848

Tribe **Auletini** Desbrochers des Loges, 1908

Subtribe **Auletina** Desbrochers, 1908

Genus ***Electrauletes*** Legalov, 2015

*E*. *unicus* Legalov, 2015 [9]–Baltic amber, Russia, Prussian Formation, Middle–late Eocene, Bartonian–Priabonian, 41.3–33.9 Ma

Subtribe **Pseudomesauletina** Legalov, 2003

Genus ***Pseudomesauletes*** Legalov, 2001

*P*. *culex* (Scudder, 1893) [58]–Florissant, USA, Florissant Formation, Uppermost Eocene, Priabonian, 34.07 ± 0.10 Ma

*P*. *ibis* (Wickham, 1912) [59]–Florissant, USA, Florissant Formation, Uppermost Eocene, Priabonian, 34.07 ± 0.10 Ma

*P*. *lobanovi* Bukejs et Legalov, 2021 [27]–Baltic amber, Russia, Prussian Formation, Middle–late Eocene, Bartonian–Priabonian, 41.3–33.9 Ma

*P*. *obliquus* (Wickham, 1913) [60]–Florissant, USA, Florissant Formation, Uppermost Eocene, Priabonian, 34.07 ± 0.10 Ma

*P*. *striaticeps* (Wickham, 1911) [61]–Florissant, USA, Florissant Formation, Uppermost Eocene, Priabonian, 34.07 ± 0.10 Ma

Genus ***Rhynchitobius*** Sharp, 1889

*Rh*. *tanyrhinus* Poinar et Legalov, 2015 [62]–Dominican amber, Dominican Republic, Lower Miocene, Burdigalian, 20–16 Ma

*Rh*. *xuthocolus* Poinar et Legalov, 2015 [62]–Dominican amber, Dominican Republic, Lower Miocene, Burdigalian, 20–16 Ma

Subtribe **Pseudauletina** Voss, 1933

Genus ***Pseudauletes*** Voss, 1922

Subgenus *Eopseudauletes* Legalov, 2007

*P*. (*E*.) *balticus* Legalov, 2022 [32]–Baltic amber, Russia, Prussian Formation, Middle–late Eocene, Bartonian–Priabonian, 41.3–33.9 Ma

Genus ***Eoropseudauletes*** Kania et Legalov, 2019

*E*. *plucinskii* Kania et Legalov, 2019 [28]–Baltic amber, Russia, Prussian Formation, Middle–late Eocene, Bartonian–Priabonian, 41.3–33.9 Ma

Subtribe *incertae sedis*

Genus ***Teretrum*** Scudder, 1893

“*T*.” *quiescitum* Scudder, 1893 [58]–Green River, USA, Green River Formation, Lower Eocene, Ypresian, 50.6–48 Ma

*T*. *primulum* Scudder, 1893 [58]–Florissant, USA, Florissant Formation, Uppermost Eocene, Priabonian, 34.07 ± 0.10 Ma

Genus ***Docirhynchus*** Scudder, 1893

*D*. *terebrans* Scudder, 1893 [58]–Florissant, USA, Florissant Formation, Uppermost Eocene, Priabonian, 34.07 ± 0.10 Ma

Genus *incertae sedis*

“*Trypanorhynchus*” *depratus* Scudder, 1893 [58]–Florissant, USA, Florissant Formation, Uppermost Eocene, Priabonian, 34.07 ± 0.10 Ma

“*Paltorhynchus*” *rectirostris* Scudder, 1893 [58]–Florissant, USA, Florissant Formation, Uppermost Eocene, Priabonian, 34.07 ± 0.10 Ma

“*Trypanorhynchus*” *sedatus* Scudder, 1893 [58]–Florissant, USA, Florissant Formation, Uppermost Eocene, Priabonian, 34.07 ± 0.10 Ma

Genus ***Paleauletobius*** Legalov, 2007

*P*. *silenus* (Heer, 1847) [63]–Öhningen, Germany, Upper Freshwater Molasse Formation, Upper Miocene, Langhian-Tortonian, 15–11.1 Ma

Tribe **Rhynchitini** Gistel, 1848

=Isotheinae Scudder, 1893

Subtribe **Temnocerina** Legalov, 203

Genus ***Eocenorhynchites*** Legalov, 2012

*E*. *vossi* Legalov, 2012 [30]–Baltic amber, Russia, Prussian Formation, Middle–late Eocene, Bartonian–Priabonian, 41.3–33.9 Ma

Subtribe **Perrhynchitina** Legalov, 2003

Genus ***Succinorhynchites*** Legalov, 2013

*S*. *alberti* Legalov, 2013 [31]–Baltic amber, Russia, Prussian Formation, Middle–late Eocene, Bartonian–Priabonian, 41.3–33.9 Ma

Genus ***Tatianaerhynchites*** Legalov, 2002

*T*. *goergesi* (Zherikhin, 1992) [48]–Rott, Germany, Rott Formation, Upper Chattian, 24.0–23.0 Ma

Subtribe **Rhynchitina** Gistel, 1848

Genus ***Cartorhynchites*** Voss, 1938

*C*. *struvei* Zherikhin, 1992 [48]–Rott, Germany, Rott Formation, Upper Chattian, 24.0–23.0 Ma

*C*. *groehni* **sp. n.** [present paper]–Baltic amber, Russia, Prussian Formation, Middle–late Eocene, Bartonian–Priabonian, 41.3–33.9 Ma

Genus ***Opacoinvolvulus*** Legalov, 2003

*O*. *rottensis* (Zherikhin, 1992) [48]–Rott, Germany, Rott Formation, Upper Chattian, 24.0–23.0 Ma

*O*. *zherichini* Legalov, 2003 [48]–Rott, Germany, Rott Formation, Upper Chattian, 24.0–23.0 Ma

Genus ***Epirhynchites*** Voss, 1969

Subgenus *Tshernyshevinius* Legalov, 2003

*E*. (*T*.) *auratus* (Scopoli, 1763)–Binagady, Azerbaijan, Middle Pleistocene; Odintsovo interglaciation, 0.190 Ma

=*Rhynchites martynovi* Ter-Minassian, 1947 [64]

Subtribe *incertae sedis*

Genus ***Isothea*** Scudder, 1893

*I*. *alleni* Scudder, 1893 [58]–Florissant, USA, Florissant Formation, Uppermost Eocene, Priabonian, 34.07 ± 0.10 Ma

Genus ***Trypanorhynchus*** Scudder, 1893

*T. corruptivus* Scudder, 1893 [58]–Florissant, USA, Florissant Formation, Uppermost Eocene, Priabonian, 34.07 ± 0.10 Ma

Genus ***Prodeporaus*** Legalov, 2003

=*Paleauletes* Legalov, 2003

*P*. *curiosum* Scudder, 1893 [58]–Florissant, USA, Florissant Formation, Uppermost Eocene, Priabonian, 34.07 ± 0.10 Ma

*P*. *exanimale* Scudder, 1893 [58]–Florissant, USA, Florissant Formation, Uppermost Eocene, Priabonian, 34.07 ± 0.10 Ma

*P*. *exilis* (Wickham, 1913) [60]–Florissant, USA, Florissant Formation, Uppermost Eocene, Priabonian, 34.07 ± 0.10 Ma

*P*. *minutissimus* (Wickham, 1913) [60]–Florissant, USA, Florissant Formation, Uppermost Eocene, Priabonian, 34.07 ± 0.10 Ma

*P*. *smithii* (Scudder, 1893) [58]–Florissant, USA, Florissant Formation, Uppermost Eocene, Priabonian, 34.07 ± 0.10 Ma

Genus ***Prodeporaides*** Legalov, 2003

“*P*.” *laminarum* (Wickham, 1916) [65]–Florissant, USA, Florissant Formation, Uppermost Eocene, Priabonian, 34.07 ± 0.10 Ma

“*P*.” *subterraneus* (Scudder, 1893) [58]–Florissant, USA, Florissant Formation, Uppermost Eocene, Priabonian, 34.07 ± 0.10 Ma

“*P*.” *vulcan* (Wickham, 1916) [65]–Florissant, USA, Florissant Formation, Uppermost Eocene, Priabonian, 34.07 ± 0.10 Ma

*P*. *wymani* (Scudder, 1893) [58]–Florissant, USA, Florissant Formation, Uppermost Eocene, Priabonian, 34.07 ± 0.10 Ma

Genus *incertae sedis*

“*Masteutes*” *saxifer* Scudder, 1893 [58]–Florissant, USA, Florissant Formation, Uppermost Eocene, Priabonian, 34.07 ± 0.10 Ma

“*Rhynchites*” *hageni* Heyden et Heyden, 1866 [66]–Rott, Germany, Rott Formation, Upper Chattian, 24.0–23.0 Ma

“*Rhysosternum*” *punctatolineatum* Piton, 1940 [67]–Menat, Middle-Upper Paleocene, Selandian-Thanetian, 61.0–59.0 Ma

Tribe **Eugnamptini** Voss, 1930

Genus ***Eugnamptidea*** Wickham, 1912

“*E*.” *florissantensis* (Wickham, 1913) [60]–Florissant, USA, Florissant Formation, Uppermost Eocene, Priabonian, 34.07 ± 0.10 Ma

*E*. *robusta* Wickham, 1916 [65]–Florissant, USA, Florissant Formation, Uppermost Eocene, Priabonian, 34.07 ± 0.10 Ma

*E*. *tertiaria* Wickham, 1912 [68]–Florissant, USA, Florissant Formation, Uppermost Eocene, Priabonian, 34.07 ± 0.10 Ma

Genus ***Eugnamptus*** Schoenherr, 1839

*E*. *proterus* Poinar et Brown, 2007 [69]–Mexican amber, Mexico, Sinojovel Formation, Lower Miocene, Burdigalian, 20–16 Ma

## 4. Discussion

The oldest Rhynchitidae, which belong to the subfamily Sanyrevilleinae, were found in the Turonian age in the USA and Botswana [25,55,56]. A representative of the tribe Rhynchitini, “*Rhysosternum*” *punctatolineatum*, was described from the Paleocene in France [34,67]. A specimen, probably in Auletini, was recorded from the early Eocene in Green River [34,58]. In total, 13 tooth-nosed snout weevils have been described from Baltic amber. Nearly half of the species [9,16,25,27] are of the genus *Baltocar* (Sayrevilleinae). Rhynchitinae are represented by seven species from the subtribes Auletina [9], Pseudomesauletina [27], Pseudauletina [28,32] of the tribe Auletini, and Temnocerina [30], Perrhynchitina [31], and Rhynchitina [given here]. The rhynchitid fauna of the terminal Eocene of Florissant is quite diverse, but the type material needs to be re-examined to clarify the systematic position of the taxa. It includes the first record of the earliest tribe, Eugnamptini [60,65,68]. Representatives of the tribes Auletini and Rhynchitini are also known from the Florissant [9,34,58,60,65].

The Oligocene tooth-nosed snout weevils were found in two Lagerstätten of Germany. An undetermined species of the tribe Rhynchitidae is illustrated in the book “Fossil Insects: an introduction to palaeoentomology” on p. 60, Figure 48 [70] from Enspel. Six species of the tribes Vossicartini (Sayrevilleinae) and Rhynchitini (Rhynchitinae) were described from the upper Chattian of Rott [9,34,48,57,66]. Four species belong to the extant genera *Tatianaerhynchites*, *Cartorhynchites,* and *Opacoinvolvulus.* Representatives of the tribe Auletini and Eugnamptini are found in Dominican [62] and Mexican amber [69] and also in the upper Miocene of Germany [63].

Quaternary Rhynchitidae are very rare. *Rhynchites martynovi* was described as a new species by Ter-Minassian [64] from the Odintsovo (=Korshov, =Lublinian) interglaciation (Middle Pleistocene, MIS-7). However, it was synonymized with the common extant species *Epirhynchites auratus* of Azerbaijan fauna [9].

**Figure 4:**
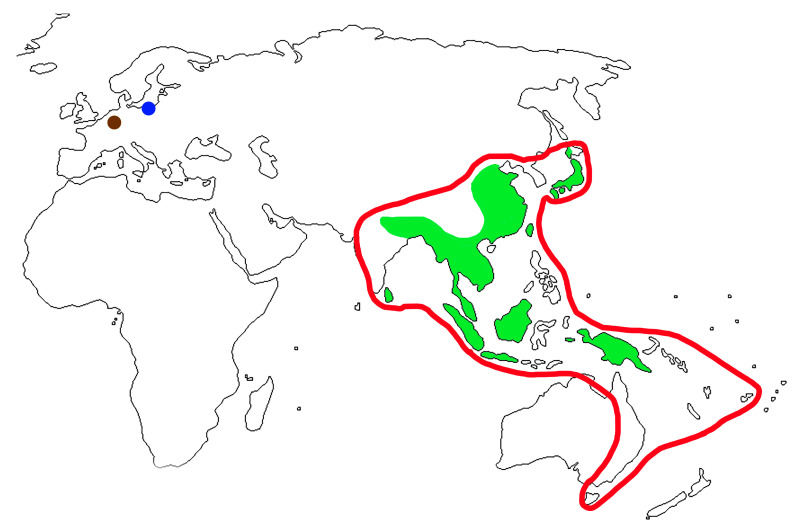
Distribution of the species of the genus *Cartorhynchites* and genus *Symplocos*: recent members of *Cartorhynchites*—green shaded area; recent distribution of *Symplocos*—red line; blue circle—Eocene record of *Cartorhynchites*; and brown circle—Oligocene record of *Cartorhynchites*.

The genus *Cartorhynchites* includes more than 30 extant and 2 extinct species [37,48,71]. Modern forms are distributed in the Oriental and Australian regions (Figure 4). Records of the genus for the south of the Russian Far East [47] are doubtful. Fossil species are known from late Eocene Baltic amber (represented by the newly described species) and the uppermost Oligocene of Germany [48]. This is an additional example confirming the links between the late Eocene fauna of Europe and the Oriental fauna as well as the previously recorded [29,33] Metrioxenini (Belidae), Rhadinocybini, and Notapionini (Brentidae, Apioninae), *Conapium* Motschulsky, 1866 (Brentidae, Apioninae, Piezotrachelini), Aedemonini (Curculionidae: Molytinae), and Stromboscerini (Curculionidae, Dryopththorinae). The presence of the genus *Cartorhynchites* in the late Oligocene suggests that the elements of modern Oriental fauna survived during the Eocene–Oligocene extinction event in Europe and possibly lived until the Pleistocene cooling. Although the genus *Cartorhynchites* was not found in the European Miocene and later, the genus *Phialodes* Roelofs, 1874, actually distributed in East Asia [37], is represented in the Miocene (Öhningen) of Germany [31,72]. A representative of the Oriental group Hoplapoderina (Attelabidae), *Phymatapoderus flavimanus* (Motschulsky, 1860), lived in Belarus during the warm period of the Pleistocene [73].

Species of the genus *Cartorhynchites* develop on different representatives of the genus *Symplocos* Jacq. (*S*. *microcalyx* Hayata, *S*. *coreana* (H. Lév.) Ohwi) from the family Symplocaceae [35,74]. The present-day distribution of the genus *Cartorhynchites* is completely situated within the range of Symplocaceae (Figure 4). *Symplocos kowalewskii* (Casp.) Sadowski et Hofmann has recently been reported in Baltic amber [75]. This extinct plant could be a suitable host plant for *Cartorhynchites groehni* sp. n. described in the current paper. *Symplocos myosotis* (Unger) Weyland was known from Rott (Oligocene) [76], and possibly *Cartorhynchites struvei* was associated with this plant.

Studies of the fossil Coleoptera fauna in Baltic amber have been particularly intensive in the last decade. An especially large amount of material has been processed, or sufficient study progress has been made for such beetle groups as, for example, the predatory Cantharidae [77,78,79,80,81,82,83,84,85,86,87,88,89,90,91,92,93,94,95,96,97,98,99,100,101,102,103,104,105,106,107,108,109,110,111,112,113] and Coccinellidae [114,115,116,117,118], as well as the herbivorous Cerambycidae [119,120,121,122,123,124,125,126,127,128,129]. New descriptions of species and genera have significantly added to the picture of the fossil beetle assemblage in the Fennosarmatian landmass during the Late Eocene and made adjustments to the conclusions about the paleoecosystems of amber forests. The recent active work on the inventory of the Baltic amber flora [50,51,75,130,131,132,133,134,135] provides a very useful tool for understanding the possible trophic habits of phytophagous beetles and for the reconstruction of possible “host plant–phytophagous beetle” associations in the Paleogene. The family Rhynchitidae is a small part of the Eocene Baltic amber assemblage, consisting of 13 species within 8 genera, but the ongoing study of tooth-nosed snout weevils is a promising addition to the understanding of the interactions between plant-feeders and vegetation as well as the late Eocene stage of insect–plant co-evolution.

## 5. Conclusions

A new extinct species of the evolutionarily advanced subtribe Rhynchitina was found in Baltic amber. Perhaps it developed on a species of *Symplocos* from the family Symplocaceae, e.g., *S. kowalewskii*, also known in Baltic amber. The discovery of a representative of the genus *Cartorhynchites* confirms the links between modern Oriental fauna and the fauna of the late Eocene of Europe.

## Data Availability

The specimen is deposited in the Center of Natural History (Centrum für Naturkunde–CeNak; formerly the Geological-Paleontological Institute and Museum–das Geologisch-Paläontologische Museum) [GPIH] of the University of Hamburg, Germany.

## Supplementary Materials

The following supporting information can be downloaded at: https://www.mdpi.com/article/10.3390/life13091920/s1.
